# Temperature Dependent Depuration of Norovirus GII and Tulane Virus from Oysters (*Crassostrea gigas*)

**DOI:** 10.1007/s12560-022-09547-8

**Published:** 2023-01-19

**Authors:** Sarah M. Stoppel, Arne Duinker, Mamata Khatri, Bjørn Tore Lunestad, Mette Myrmel

**Affiliations:** 1 Section for Seafood Hazards, Institute of Marine Research, Bergen, Norway; 2 Faculty of Veterinary Medicine, Norwegian University of Life Sciences, Ås, Norway

**Keywords:** Norovirus, Tulane virus, Pacific oyster, Depuration, Temperature, Infectivity

## Abstract

Raw oysters are considered a culinary delicacy but are frequently the culprit in food-borne norovirus (NoV) infections. As commercial depuration procedures are currently unable to efficiently eliminate NoV from oysters, an optimisation of the process should be considered. This study addresses the ability of elevated water temperatures to enhance the elimination of NoV and Tulane virus (TuV) from Pacific oysters (*Crassostrea gigas*). Both viruses were experimentally bioaccumulated in oysters, which were thereafter depurated at 12 °C and 17 °C for 4 weeks. Infectious TuV and viral RNA were monitored weekly for 28 days by TCID_50_ and (PMAxx-) RT-qPCR, respectively. TuV RNA was more persistent than NoV and decreased by < 0.5 log_10_ after 14 days, while NoV reductions were already > 1.0 log_10_ at this time. For RT-qPCR there was no detectable benefit of elevated water temperatures or PMAxx for either virus (*p* > 0.05). TuV TCID_50_ decreased steadily, and reductions were significantly different between the two temperatures (*p* < 0.001). This was most evident on days 14 and 21 when reductions at 17 °C were 1.3–1.7 log_10_ higher than at 12 °C. After 3 weeks, reductions > 3.0 log_10_ were observed at 17 °C, while at 12 °C reductions did not exceed 1.9 log_10_. The length of depuration also had an influence on virus numbers. TuV reductions increased from < 1.0 log_10_ after seven days to > 4.0 log_10_ after 4 weeks. This implies that an extension of the depuration period to more than seven days, possibly in combination with elevated water temperatures, may be beneficial for the inactivation and removal of viral pathogens.

**Supplementary Information:**

The online version contains supplementary material available at 10.1007/s12560-022-09547-8.

## Introduction

Shellfish are commonly viewed as culinary delicacies, giving high retail prices. This is especially true for bivalve molluscs. Among those, raw oysters are the pinnacle of fine dining. Still, this high-class product is often associated with food-borne illness. In this context, norovirus (NoV) is of special relevance, as it is perceived as the most common cause of non-bacterial gastroenteritis globally. Even though NoV is known to spread directly from person to person, transmission through food is common. Shellfish, predominantly oysters, are repeatedly listed as the culprit in food-borne NoV infections (Bellou et al., 2013; Bitler et al., 2013; Guix et al., 2019; Hardstaff et al., 2018), and the occurrence of NoV outbreaks after the consumption of oyster dishes is well-documented (Baker et al., 2011; Iritani et al., 2014; Meghnath et al., 2019; Woods et al., 2016).

A widespread practice to remove pathogens is keeping oysters in tanks with clean sea water in a process termed depuration (Lee et al., 2008; McLeod et al., 2017b). This process has been shown to work well for the removal of bacterial pathogens. However, depuration is less effective for NoV, and outbreaks have repeatedly been linked to depurated oysters (McLeod et al., 2017a; Neish, 2013; Sharp et al., 2021; Shen et al., 2019). Allegedly, this is due to the binding of NoV particles to carbohydrate receptors present in the oyster digestive tissue (DT). These receptors closely resemble histo-blood group antigens (HBGAs) in the human intestine, to which NoV has been shown to bind (Huang et al., 2003; Le Guyader et al., 2006; Marionneau et al., 2002; Tian et al., 2006).

As commercially applied depuration is unable to efficiently prevent NoV infections associated with oyster consumption, modifications to the depuration process have been evaluated. The effects of light regimen, feeding, salinity and vibration have been examined. Also, depuration times have been extended for up to 6 weeks, and a promising approach may be elevated water temperatures (Battistini et al., 2021b; Drouaz et al., 2015; Kingsley et al., 2018; Martinez-Albores et al., 2020; Neish, 2013; Polo et al., 2018; Rupnik et al., 2021; Younger et al., 2020). A minimum of 8 °C is recommended for the depuration of Pacific oysters in the UK, but temperatures can vary depending on location. In China and parts of Australia water temperatures can even reach 25 °C (McLeod et al., 2017b). There is evidence that raising the water temperature increases the elimination of bacteria (Chae et al., 2009). As for virus removal, a benefit of elevated temperatures has also been suggested, but this may depend on the type of virus (Neish, 2013; Rupnik et al., 2021; Younger et al., 2020).

In the present study, a NoV GII.2 strain and its culturable surrogate Tulane virus (TuV) have been bioaccumulated in the Pacific oyster, *Crassostrea gigas*. This bivalve is invasive to Scandinavia and is considered both an intruder and a potential food resource. Contaminated oysters were placed in depuration tanks at two water temperatures to assess whether elevated temperatures improve the elimination of the two viruses. Infectious TuV as well as persistence of TuV and NoV RNA were monitored weekly over the course of 28 days.

## Materials & Methods

### Viruses & Cells

TuV strain M033 was provided by T. Farkas, Louisiana State University at Baton Rouge, LA, United States. LLC-MK2 cells (ATCC CCL-7) were grown in cell culture flasks with filter cap in Medium 199/Earle's salts/GlutaMAX (Thermo Scientific/Gibco, Waltham, MA, USA) with 10% foetal bovine serum (Thermo Scientific/Gibco) and 2% antibiotic/antimycotic solution (Thermo Scientific/Gibco) at 37 °C and 5% CO_2_. Cells were infected with TuV at a multiplicity of infection of 1 in M199 Earle's salts/GlutaMAX without additional supplements (maintenance medium). When a cytopathic effect was observed, TuV was harvested by 3× freeze-thawing. Debris was removed by centrifugation at 2500×*g* for 5 min. The supernatant was collected, and the titre of virus stock determined (3.1 × 10^7^ TCID_50_/mL). The NoV GII.2 faecal sample was acquired from a child with acute gastroenteritis. The genotype was determined based on ORF2 sequencing and the Norovirus Automated Genotyping Tool (Kroneman et al., 2011).

### Oysters

Oysters harvested near Nøtterøy, Vestfold and Telemark County, Norway, were provided by a local supplier. Oysters were size graded by the supplier and weighed 120 g on average.

### Bioaccumulation and Depuration

Bioaccumulation was conducted in late June 2021 in natural sea water that was treated by UV irradiation. TuV and NoV stock was diluted 1:1000 in 60 L sea water to 3.1 × 10^4^ TCID_50_/mL and 4.0 × 10^3^ genome copies per millilitre (GC/mL), respectively. Two trays, each containing ~ 75 oysters were placed in tanks with sea water so that all were completely submerged. Bioaccumulation lasted for 20 h at a water temperature of ~ 12 °C and under constant aeration of the water. There was no feed added to the bioaccumulation tank. Oysters were rinsed in clean sea water and 15 oysters were collected to determine the initial virus concentration. The remaining oysters were divided equally and placed into two depuration tanks that differed in water temperature. Low temperature depuration was conducted at 12 ± 0.9 °C using incoming sea water routinely used in the depuration facility. Elevated temperature depuration was at 17 ± 0.2 °C. The water temperature was held stable by a swimming pool heat pump (Heat Splasher ECO Plug & Play 2,5 kW, model 1295). Depuration lasted for 4 weeks in a flow-through system without water recirculation.

### Sample Processing and Virus Extraction

Samples were collected weekly until day 28. From each depuration tank at each sampling time, 15 oysters were collected, rinsed in clean sea water, and transported chilled to the laboratory within 3 h. Oysters were placed at 4 °C upon arrival for a maximum of 2 h until analysis. Oysters were shucked, DT removed and homogenised with scalpel blades. DT from five oysters was pooled to make one sample, resulting in three replicates per depuration temperature per sampling day. Virus was extracted from oyster tissue as described in Araud et al. (2016) with minor modifications. Homogenised tissue was aliquoted into 50 mL tubes and 5 mL PBS was added. The tissue was further homogenised with a pestle. Samples were centrifuged at 3000×*g* for 15 min. The supernatant was collected and immediately used for molecular and cell culture analysis. Excess virus extract was stored at − 80 °C.

### 50% Tissue Culture Infective Dose (TCID_50_)

LLC-MK2 cells were seeded into 96-well plates (~ 1 × 10^4^ cells/well) and grown to confluency. Virus extracts were diluted ten-fold in maintenance medium. Fifty microlitre of these virus dilutions was inoculated into the wells in quadruplicate for incubation at 37 °C and 5% CO_2_ for 2 h. Maintenance medium (200 µL) was added, and plates were incubated at 37 °C and 5% CO_2_ until 7 days post infection. Cytopathic effect was converted to TCID_50_ per mL virus extract with the Spearman & Kärber algorithm described by Hierholzer and Killington (1996).

### Propidium Monoazide (PMAxx) Treatment

PMAxx was included in the analysis to reduce PCR amplification of viruses with damaged capsids (Randazzo et al., 2018; Razafimahefa et al., 2021). PMAxx (20 mM, Biotium, Fremont, CA, USA) was diluted 1:10 in diH_2_O and added to 500 µL virus extract at a final concentration of 100 µM. Samples were incubated with shaking (150 rpm) at room temperature for 15 min in the dark. They were exposed to LED light (464–476 nm) for 15 min in the PhastBlue Photoactivation System for Tubes (GenIUL, Terrassa, Spain) at 100% intensity and placed on ice. Virus extracts that did not receive PMAxx treatment were kept on ice.

### RNA-Extraction

RNA was isolated from 500 µL supernatant with NucliSens magnetic extraction reagents on a MiniMAG (BioMerieux, Marcy l'Etoile, France) as described in ISO 15216–1 (ISO, 2017). RNA was used for molecular analysis immediately after extraction or stored at − 80 °C.

### One-Step RT-qPCR

The RNA UltraSense One-Step Quantitative RT-PCR System (Thermo Scientific/Applied Biosystems) was used at a reaction volume of 20 µL. TVIF primer and probe were chosen for TuV and COG2R/QNIF2d primers and QNISP probe for GII (Table 1). TVIF primer and probe concentrations were 0.4 and 0.05 µM, respectively. Concentrations of QNIF2d, COG2R and QNIFSP were 0.9, 0.5 and 0.25 µM. Cycling conditions were 55 °C/30 min, 95 °C/2 min, and 45 cycles of 95 °C/15 s, 55 °C/20 s and 64 °C/40 s for TuV. NoV was run at 55 °C/30 min, 95 °C/2 min, and 45 cycles of 95 °C/15 s, 60 °C/60 s, 64 °C/8 s and 68 °C/8 s.

**Table 1:** Oligonucleotides. Primer and probe sequences used in this study are listed, as well as product length and the position of the amplicon on the TuV and NoV GII genome

Oligonucleotide	Sequence (5′-3′)	Product length/position
TVIF_F	CTGGGATACCCACAACATC	107 bp/3775–3882 bp
TVIF_R	GCCAGTTAACAGCTTCAGC	
TVIF_P	FAM-GTGTGTGCCACTGGATAGCTAGCACC-BHQ	
TVIF_P.2	FAM-TGTGTGCCACTGGATAGCTAGCACC-ZEN	
COG2R	ATGTTCAGRTGGATGAGRTTCTCWGA	88 bp/5012–5100 bp
QNIF2d	TCGACGCCATCTTCATTCACA	
QNIFSP	JOE-AGCACGTGGGAGGGCGATCG-BHQ	
QNIFSP.2	JOE-AGCACGTGGGAGGGCGATCG-ZEN	

TVIF sequences were taken from Drouaz et al. (2015). NoV primer and probe sequence are found in Kageyama et al. (2003) and Loisy et al. (2005). Probes were modified for the RT-ddPCR assays.

### Droplet Digital RT-PCR (RT-ddPCR)

For quantification of genome copies (GC) per mL after RT-qPCR, NoV and TuV positive controls were quantified in RT-ddPCR. The One-Step RT-ddPCR Advanced Kit for Probes (Bio-Rad, Hercules, CA, USA) and the QX200 Droplet Digital System (Bio-Rad) were used. The NoV stock was determined to be 4.0 × 10^6^ GC/mL according to the method described by Persson et al. (2018). QNIFSP and TVIF probes were modified with a ZEN/Iowa Black FQ quencher (Table 1). Primer and probe concentrations were adjusted to 0.9 and 0.25 µM, respectively. TuV cycling conditions were 48 °C/60 min, 95 °C/10 min, and 39 cycles of 95 °C/30 s and 55 °C/60 s with a ramp speed of 2 °C/s, and a final elongation step of 98 °C/10 min. For NoV, annealing temperature was reduced to 53 °C and ramp speed was 3 °C/s. The QuantaSoft™ software (Bio-Rad), was used for data analysis and the accepted number of droplets generated was ≥ 10,000.

### Data Analysis

The experiment was conducted once in summer 2021. Three biological replicates were analysed at each sampling point. RT-qPCR was run on technical duplicates. Relative viral RNA copy numbers were estimated using standard curves from ten-fold dilution series of stock virus RNA. Copy numbers were expressed with the following formula: ${N}_{1}={N}_{2}\times {\left(1+E\right)}^{\left({Ct}_{2}-{Ct}_{1}\right)}$. N_1_ and N_2_ are the amount of viral RNA in the sample and in the positive control, respectively; Ct_1_ and Ct_2_ are threshold cycles for sample and control, respectively; E is the efficiency of amplification. These numbers were the basis for calculating log_10_ reductions as follows: log_10_ reduction = log_10_ (control virus) – log_10_ (inactivated virus). ANOVA and Tukey HSD post-hoc test was conducted in RStudio version 1.3.959 (R Core Team, 2018; RStudio Team, 2020). *P* values < 0.05 were deemed significant.

## Results

### Persistence of Viral RNA (TuV, NoV)

**Fig. 1:**
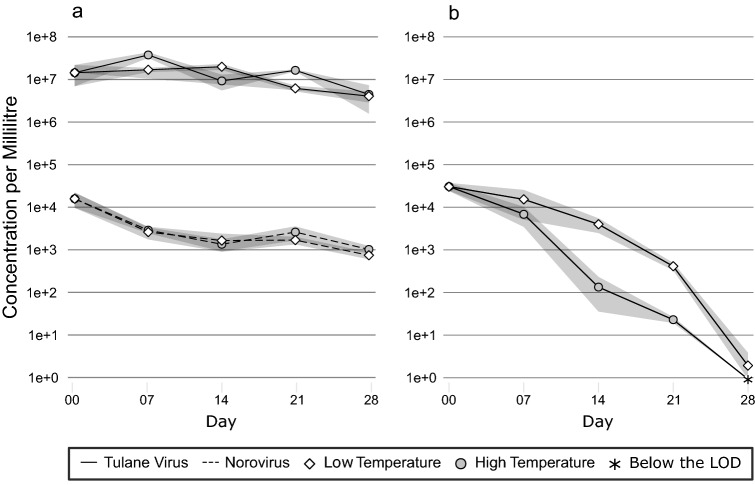
Concentration of TuV (solid line) and NoV (dashed line) RNA in genome copies per mL as determined by RT-qPCR without the addition of PMAxx (**a**), as well as TuV titre in TCID_50_ per mL (**b**) during the depuration period at high (filled circle) and low (empty diamond) water temperatures; the ribbon represents standard error of three replicates,  * (asterisk) = Below the limit of detection (LOD)

RT-qPCR underestimated the reduction in infectious TuV compared to TCID_50_ (Fig. 1a, b). TuV RNA decreased by < 1.0 log_10_ after 28 days. After 3 weeks, a TuV reduction of ~ 0.3 log_10_ was observed at low water temperature. At the higher water temperature, a 0.3 log_10_ reduction in TuV RNA occurred one week earlier. Still, no significant difference in reduction could be detected between the two temperatures (*p* > 0.05).

NoV GII decreased by 0.8 log_10_ after week one (Fig. 1a). Prolonged depuration (≥ 14 days) resulted in a ≥ 1.0 log_10_ reduction in NoV. Over the course of the depuration period, an average reduction of 1.3 log_10_ was observed, but there was no significant difference in reduction between the two temperatures (*p* = 0.057).

There was no observable benefit of applying PMAxx for differentiating between infectious and non-infectious virus for either TuV or NoV (*p* > 0.05, data not shown). PMAxx data is available as a supplementary file online.

### Reduction in Infectious Virus (TuV)

TuV titre was monitored throughout the 4-week depuration period. The initial TuV concentration was 3.0 × 10^4^ TCID_50_ per mL supernatant (Fig. 1b). Regardless of water temperature, TuV decreased by < 0.8 log_10_ within the 1st week to 6.7 × 10^3^ and 1.5 × 10^4^ TCID_50_/mL at high and low temperatures, respectively. Towards the end of the experiment, a reduction of ~ 4.2 log_10_ to < 10 TCID_50_/mL was recorded at 12 °C, while at 17 °C TuV was reduced by 4.5 log_10_ to below the limit of detection. Overall, there was a significant difference in reduction of infectious TuV at high and low temperature (*p* < 0.001). This was most evident on days 14 and 21 (*p* < 0.001, *p* = 0.005).

## Discussion

Depuration has shown a limited ability to remove NoV from oysters. One important factor influencing virus removal rates may be water temperature during depuration. Lower temperatures lead to low metabolic activity in oysters and seem to decrease virus removal rates (Lees et al., 2010). Therefore, the elimination of infectious viruses may increase at higher water temperatures.

Our data suggest a significant effect of the elevated water temperature on infectious TuV from week 2 onwards. TCID_50_ reductions on day 14 were 2.6 and 1.0 log_10_ at 17 °C and 12 °C, respectively. After 3 weeks, reductions > 3.0 log_10_ were observed at the higher water temperature, while at the lower temperature reductions were still < 2.0 log_10_. After 4 weeks, no infectious TuV was detected at 17 °C, but small numbers of infectious TuV remained at the low temperature. The benefit of elevated temperatures during depuration for the reduction in virus infectivity has been illustrated previously (Kingsley et al., 2018; Neish, 2013).

Contrary to TuV infectivity, we found no advantage of the higher temperature for the reduction in either TuV or NoV RNA. Correspondingly, Polo et al. (2018) report that TuV RNA levels in Pacific oysters were comparable at temperatures between 9 and 17 °C. Still, for the reduction in NoV RNA, a beneficial effect of elevated temperatures has been suggested (Choi & Kingsley, 2016; Neish, 2013; Rupnik et al., 2021; Younger et al., 2020). It is conceivable that a greater difference in water temperature results in a greater difference in depuration kinetics (Rupnik et al., 2021). Our experiment was conducted in June to July, so the sea water temperature was already ≥ 11.5 °C. The ambient water temperature was kept as is after entering the depuration facility, a common practice for the supplier, and was directly used for the low temperature tank. To get an impression of the effect of elevated water temperatures during colder months, additional data should be gathered in winter to complement present results. During this time of year NoV is more prevalent in oysters (EFSA, 2019) and sea water temperatures can fall below 5 °C in Scandinavian waters.

Not only did the elevated water temperature have no effect on the removal of virus RNA, but RNA levels remained comparatively stable throughout the 4-week trial. Likewise, virus RNA decreased within the 1st days and no benefit of prolonged depuration for the reduction in *Caliciviridae* RNA was observed in related work (Battistini et al., 2021b; Choi & Kingsley, 2016; Rupnik et al., 2021; Ueki et al., 2007). Similarly, we observed the highest reductions in NoV RNA within the 1st week. For the remainder of the experiment, NoV levels were rather stable. A decrease in RNA during the 1st week and no noticeable reduction afterwards may reflect that unbound NoV was washed out of the oysters briefly after bioaccumulation, whereas bound virus remained within oysters for the rest of the study. Conversely, a continuous decline in TuV and NoV GI RNA could be demonstrated by Drouaz et al. (2015) and Polo et al. (2018). The stability of TuV RNA during our depuration trial, contrary to the steady decrease in infectious TuV, may indicate virus inactivation in the oyster without significant virus elimination, as previously suggested by Leduc et al. (2020). In contrast, Polo et al. (2018) detected similar reductions in TuV infectivity and RNA, which implies TuV elimination. These discrepancy underlines the importance of consulting data on virus infectivity in addition to RT-qPCR data. To come to a consensus on the fate of TuV in oysters, further studies are needed.

Just like there was no advantage of the elevated water temperature on RNA removal, we did not find any effect of PMAxx for either virus. However, application of the dye has been advantageous over regular RT-qPCR in distinguishing infectious from non-infectious NoV in oysters (Randazzo et al., 2018). Randazzo et al. (2018) examined the effect of PMAxx on heat treated virus, and they extracted NoV according to the ISO standard, which includes incubation at 37 °C and 60 °C (ISO, 2017). These temperatures would have facilitated the entry of PMAxx due to induced capsid damage. During depuration in the present study the capsid of TuV and NoV was most likely not exposed to conditions that induce severe capsid damage. Accordingly, infectious TuV declined in TCID_50_, whereas no effect on the RNA or of PMAxx was observed.

In the present work we also observed a more rapid decline in NoV compared to TuV RNA during the 1st week. NoV RNA was already reduced by ~ 0.8 log_10_ after the 1st week, whereas TuV RNA was reduced by < 0.8 log_10_ at the end of the experimental period. Several factors may contribute to this, among them the conditions encountered during bioaccumulation or a difference in the efficiency of virus recovery from oyster tissue. We performed the bioaccumulation of both viruses simultaneously, so there may have been competition over HBGA-binding. TuV may have outcompeted NoV in binding since TuV concentration during bioaccumulation exceeded that of NoV. Additionally, TuV may have a greater binding affinity than NoV GII.2. As a result, the majority of NoV may have been present as an unbound fraction in the oyster DT and would have been eliminated quickly. In oysters, Ueki et al. (2021) report unsuccessful bioaccumulation of GII.2, whereas Langlet et al. (2015) confirmed at least a low binding capacity to homogenised oyster tissue. Moreover, seasonal variations in NoV binding to oyster tissue were demonstrated by Maalouf et al. (2010). They suggest that HBGA expression is higher during late winter and spring. As mentioned, the present study was conducted in mid-summer, so that HBGA expression in oyster DT may have been decreased and might have influenced virus binding. Nevertheless, NoV must have bound to receptors, as NoV RNA was detected throughout the experiment. A possible difference in binding affinity could be further investigated and a different NoV strain could be chosen for future work.

The reductions in NoV RNA observed in the present study may result in a safe product, but this will depend on the initial level of NoV contamination. According to the European Food Safety Authority (EFSA) NoV contamination levels in oysters averaged at 337 GC/g, and only 5.5% of samples were > 1000 GC/g (EFSA, 2019). In the present depuration trial, ~ 0.8 and 1.0 log_10_ of the initial NoV load was removed after 1 and 2 weeks, respectively. According to these reductions and the EFSA data, NoV could be reduced to ≤ 100 GC/g during a 2 week depuration. Such a low concentration in oyster DT has not been involved in NoV outbreaks according to Lowther et al. (2012). If we assume that the reduction in NoV infectivity follows a similar pattern as TuV, a 1.0 to 2.6 log_10_ decrease in infectious NoV might be expected after 14 days, depending on water temperature. Such reductions would reduce the risk of NoV infections, if NoV contamination levels are as low as the average value stated in EFSA (2019). While these numbers provide an estimate to work with, the depuration rates may vary with NoV levels and with the amount of time that has passed since contamination. In case of recent contamination most NoV may be unbound and therefore easier and faster to remove, whereas NoV that was present in the tissue for a longer time may be bound to HBGAs and thus more difficult to eliminate. In addition, EFSA (2019) determined that 1.2% of analysed samples contained > 5000 NoV GC/g, which may be encountered after recent contamination or during winter (Battistini et al., 2021a). In that case, the reductions observed in the present study may not suffice to ensure a safe product, and depuration may have to be extended to 1 month, as we observed a > 4.0 log_10_ reduction in infectious TuV after this time. The reductions in infectious TuV and NoV in oysters should be further examined, for instance by utilising the human intestinal enteroid system (Ettayebi et al., 2016) or human volunteer studies (Richards, 2012), to make sure the two viruses are comparable.

Overall, we observed an advantage of elevated water temperatures for the reduction in infectious TuV. This was noticeable after 14 days when TuV was reduced by 1.0 log_10_ and 2.6 log_10_ at the low and elevated temperature, respectively. After 7 days, reductions were not noticeably different. This implies elevated water temperatures may primarily be relevant for a 2- to 3-week depuration. After 28 days, we detected reductions in TuV infectivity of > 4.0 log_10_, regardless of temperature. In case of suspected recent faecal contamination and high virus concentrations, an extended depuration of 3 to 4 weeks could be considered. The economic burden of prolonging the depuration period must be weighed against the burden of heating up the depuration water.

## Supplementary Information

Below is the link to the electronic supplementary material.

Supplementary file1 (XLSX 53 kb)

## Data Availability

The datasets generated and/or analysed during the current study are available from the corresponding author on reasonable request.
